# HIPK2 Phosphorylates the Microtubule-Severing Enzyme Spastin at S268 for Abscission

**DOI:** 10.3390/cells8070684

**Published:** 2019-07-05

**Authors:** Alessandra Pisciottani, Loredana Biancolillo, Manuela Ferrara, Davide Valente, Francesca Sardina, Laura Monteonofrio, Serena Camerini, Marco Crescenzi, Silvia Soddu, Cinzia Rinaldo

**Affiliations:** 1 Institute of Molecular Biology and Pathology (IBPM), National Research Council (CNR), c/o Sapienza University, 00185 Rome, Italy; 2 Unit of Cellular Networks and Molecular Therapeutic Targets; IRCCS-Regina Elena National Cancer Institute, 00144 Rome, Italy; 3 Core Facilities, Italian National Institute of Health, 00161 Rome, Italy

**Keywords:** HIPK2, spastin, abscission, midbody, phosphorylation

## Abstract

Abscission is the final step of cell division, mediating the physical separation of the two daughter cells. A key player in this process is the microtubule-severing enzyme spastin that localizes at the midbody where its activity is crucial to cut microtubules and culminate the cytokinesis. Recently, we demonstrated that HIPK2, a multifunctional kinase involved in several cellular pathways, contributes to abscission and prevents tetraploidization. Here, we show that HIPK2 binds and phosphorylates spastin at serine 268. During cytokinesis, the midbody-localized spastin is phosphorylated at S268 in HIPK2-proficient cells. In contrast, no spastin is detectable at the midbody in HIPK2-depleted cells. The non-phosphorylatable spastin-S268A mutant does not localize at the midbody and cannot rescue HIPK2-depleted cells from abscission defects. In contrast, the phosphomimetic spastin-S268D mutant localizes at the midbody and restores successful abscission in the HIPK2-depleted cells. These results show that spastin is a novel target of HIPK2 and that HIPK2-mediated phosphorylation of spastin contributes to its midbody localization for successful abscission.

## 1. Introduction

Abscission is the final step of cytokinesis and consists of an orderly sequence of dynamic events that allow the final separation of the two nascent daughter cells, safeguarding the correct distribution of genomic and cytoplasmic materials in cell division [1,2]. At the end of cytokinesis, several membrane remodeling factors of the endosomal sorting complex required for transport-I (ESCRT-I) and -III pathways, such as Alix and CHMPs proteins, and the microtubule (MT)-severing enzyme spastin are sequentially recruited at the midbody, the organelle-like platform at the intercellular bridge [3]. In particular, CHMP1B recruits spastin, whose activity results in MT clearance leading to the physical separation of the two daughter cells [4,5,6]. This process depends on dramatic rearrangement of the cytoskeleton and membrane remodeling occurring at the midbody. These events are finely orchestrated by several kinases, such as Aurora-B, PLK1, and Citron kinase, which act on multiple targets to regulate the local levels, the interactions, and the functions of key players of cytokinesis [3,7,8].

Spastin belongs to the family of the AAA (associated with diverse cellular activities) ATPases. The ATPase domain (342–599 aa) is preceded by the MT binding domain (MTBD, 270–328 aa) and the MT-interacting and endosomal-trafficking domain (MIT, 116–194 aa) that is necessary for midbody recruitment through CHMP1B interaction [4,5]. Four spastin isoforms have been identified, generated by two alternative start codons (full-length M1 and the shorter isoform lacking the first 86 aa, M87) and differential splicing of the exon 4, M1∆4, and M87∆4, respectively [6,9]. M87 is ubiquitously expressed, whereas M1 expression appears to be restricted to neuronal cells [6,9,10]. Spastin has been reported to localize at regions of active MT remodeling and its depletion results in strongly delayed abscission, associated with aberrant midbody phenotype [5,11,12]. In interphase, spastin shows a diffuse staining in the nucleus and a punctate pattern in the cytoplasm, where it localizes at the centrosome, endoplasmic reticulum, endosomes, and lipid droplets [13,14,15]. Instead, during cell division, spastin localizes at the spindle poles, at the resealing nuclear envelope, and at the midbody [5,15,16].

HIPK2 is a highly conserved multifunctional tyrosine-regulated serine/threonine kinase [17,18,19], which phosphorylates a large number of targets, contributing to the fine-tuning of several pathways [20,21]. We previously observed that HIPK2-null or -depleted cells undergo cytokinesis delay and failure, accumulating elongated midbodies and tetraploid cells [22,23]. During cytokinesis, HIPK2 is recruited at the midbody in an Aurora-B-dependent manner [24] and, there, phosphorylates the extrachromosomal histone H2B at S14 [22]. This phosphorylation is not required for the midbody localization of H2B, but it is necessary for successful cytokinesis [22,24], supporting the inclusion of HIPK2 among the kinases that regulate cytokinesis. Since these kinases usually work on multiple targets, we searched for additional cytokinesis substrates of HIPK2. Here, we identify spastin as a novel abscission target of HIPK2 and show that HIPK2-mediated phosphorylation of spastin at S268 is required for the midbody localization of spastin to drive abscission.

## 2. Materials and Methods

### 2.1. Cell Culture Conditions and Expression Vectors

HeLa, U2OS, and A549 cells were cultured at 37 °C and 5% CO_2_ in DMEM GlutaMAX, supplemented with 10% heat-inactivated fetal bovine serum (FBS) (Life Technologies, Carlsbad, CA, USA); they were enriched in telophase as in [22]. The following plasmids were employed: Flag-myc empty vector (pCMV6-Entry) and flag-myc-tagged spastin-expressing vectors from OriGene Technologies (Rockville, MD, USA). Spastin mutants were obtained by site-directed mutagenesis in the Spastin-flag-myc-tagged vector using a QuikChange Lightening Kit (Agilent, Cernusco sul Naviglio, Italy) and analyzed by sequencing. Vectors were transfected by using Lipofectamine LTX and Plus reagent (Life Technologies).

### 2.2. RNA Interference (RNAi) and Real-Time (Reverse Transcription)-PCR (Real-Time (RT)-PCR)

HIPK2 RNAi was obtained by using human-specific validated stealth siRNAs (a mix of three different siRNAs by Life Technologies, as in [22]). Spastin RNAi was obtained by using a mix of specific validated stealth siRNAs by Life Technologies, targeting sequences common to all spastin isoforms. siRNAs were transfected using Lipofectamine RNAi MAX (Life Technologies). RNA extraction and real-time (RT)-PCR were performed as in [22], and relative fold-change were determined by the 2-ΔΔCt method using GAPDH mRNA as normalizer. Primers for amplification were as in [22]. SiRNA sequences were the following:

siHIPK2#1: CCCGAGUCAGUAUCCAGCCCAAUUU;
siHIPK2#2: CCACCAACCUGACCAUGACCUUUAA;
siHIPK2#3: CAGGGUUUGCCUGCUGAAUAUUUAU;
siSpastin#1: CCAGUGAGAUGAGAAAUAUUCGAUU;
siSpastin#2: CCAGUGAGAUGAGAAAUAUUCGAUU.

### 2.3. Western Blot (WB) and Co-Immunoprecipitation (co-IP)

For WB, total cell extracts (TCEs) were prepared in RIPA buffer (50mM Tris-HCl (pH 8), 600 mM NaCl, 0.5% sodium deoxycholate, 0.1% SDS, 1% NP40 and 1mM EDTA) supplemented with protease and phosphatase inhibitors (Roche, Basel, Switzerland). For co-IP, TCEs were prepared in non-denaturating lysis buffer (50 mM Tris-HCl (pH 8), 150 mM NaCl, 1% NP40 and 5 mM EDTA), supplemented with protease and phosphatase inhibitors (Roche, Basel, Switzerland). Proteins were resolved by SDS-PAGE using Bolt Novex Bis-Tris Gels 4%–12% (Life Technologies). Immunoreactivity was determined using ECL-Prime (Amersham, Buckinghamshire, UK), image acquisition and densitometric analysis were performed with Image Lab software (BIORAD, Hercules, CA, USA). The following antibodies (Abs) were employed: anti-HIPK2 (rat monoclonal Ab C5C6, kindly provided by Dr. L. Schmitz); anti-GST (1:500), anti-GAPDH (1:1000), anti-His tag (1:200), and anti-spastin (1:100; sp311/1 mouse monoclonal Ab), all provided by Santa Cruz Biotechnology, Dallas, TX, USA; anti-alpha-tubulin (1:1000; Immunological Science, Rome, Italy); anti-Flag (mouse monoclonal Ab, TA50011, OriGene Technologies, and rabbit polyclonal Ab provided by Sigma-Aldrich, St. Louis, MO, USA); anti-GFP (mouse monoclonal Ab provided by Roche and rabbit polyclonal Ab provided by Santa Cruz Biotechnology); and anti-HRP-conjugated goat anti-mouse,-rat, and -rabbit (Cell Signaling Technology, Danvers, MA, USA).

### 2.4. Kinase Assay and Recombinant Proteins

In vitro kinase assays were performed as in [19], by using HIPK2 Kinase domain (kind gift of Dr. Montemiglio) as the enzymatic source and the following recombinant proteins as substrates: His_6_-tagged spastin 79–310 (ag18866, Proteintech, Rosemont, IL, USA) and spastin M1 (TP320458, OriGene technologies).

### 2.5. GST Pull-Down and Binding Assay

GST pull-down and binding assay were performed as in [24]. In particular, GST and GST-HIPK2 were obtained by H1299 T7-vaccinia virus infection followed by transfection as described in [22] and were incubated with 100 ng of recombinant His6-tagged spastin 79–310 (Proteintech) in 50 mM Tris-HCl, pH 7.5, 150 nM NaCl. Bound Spastin was analyzed by WB.

### 2.6. Mass Spectrometry (MS)

In vitro kinase assays were performed with cold ATP, the products were resolved by NuPAGE 4%–12% (Life Technologies), and stained with the Colloidal Blue Staining kit (Life Technologies). The stained bands were cut from the gel and in gel digested with trypsin. The peptide mixture was analyzed by nanoflow-reversed-phase liquid chromatography tandem MS using an HPLC Ultimate 3000 (DIONEX, Sunnyvale, CA, USA) connected in line with a linear ion trap (LTQ, ThermoElectron, Waltham, MA, USA). MS/MS spectra were acquired in data-dependent mode, allowing the fragmentation of the five most intense ions detected in the full scan and an additional fragmentation when occurring a neutral loss of 49 or 32 Dalton.

### 2.7. p-S268 Spastin Ab Production and Purification

Rabbit immunization was performed with the following amide-conjugated phosphopeptide SGHHRAP(pS)YSGLSMV. The obtained serum was positive/negative affinity, purified by using phosphorylated/non-phosphorylated peptides (Life Technologies).

### 2.8. Immunofluorescence (IF) and Quantitative Analysis of Signals

Cells were seeded onto poly-L-lysine-coated coverslips, fixed in 2% formaldehyde or in ice-cold methanol, permeabilized in 0.25% Triton X-100 in PBS for 10 min, and then blocked in 5% bovine serum albumin in PBS before the primary Ab was applied. For each employed Ab and IF condition see Supplementary Table S1. Appropriate secondary FITC- or TRITC-conjugated Abs (Alexa-fluor, Life Technologies) were used. DNA was marked with DAPI (Sigma-Aldrich). Preparations were examined under an Olympus AX70 microscope using a 100×/1.35 NA objective. Images for each sample were taken in parallel using identical microscope settings. Signals were measured by using Image J software. Quantification of IF signals were performed as follows: a) p-S268 spastin at the midbody: mean of pixel intensity at midbody corrected for external background; and b) for MT density quantification, β-tubulin fluorescence intensity was measured by drawing cellular outline and measuring the mean of pixel intensity corrected for external background.

### 2.9. MT Depolymerization Assay

Cells were placed on ice for indicated times in the presence of the MT depolymerizing agent 10 μM nocodazole (Sigma-Aldrich), fixed and then processed by IF. Hyperstable MTs were visualized by using anti-acetylated-tubulin.

### 2.10. Statistics

Statistical analyses were performed using the GraphPad Prism software (GraphPad Software Inc., CA, USA). Data were tested for normality using Shapiro–Wilk normality test. The unpaired *t*-test or Chi-square (χ²test) was applied to determine the significance of quantitative experiments when the data distribution was normal. The Mann–Whitney test was used when the populations did not show a Gaussian distribution. Statistical significance was set at *p* < 0.05.

## 3. Results

### 3.1. Spastin is Not Detectable at the Midbody in HIPK2-Depleted Cells

To identify new HIPK2 targets in cytokinesis, we examined the midbody localization of several structural and functional cytokinesis factors at the single-cell level by IF. Previous works have shown that acute HIPK2 depletion results in stronger cytokinesis defects when compared with chronic HIPK2 depletion, due to compensatory events [22,23].

**Figure 1:**
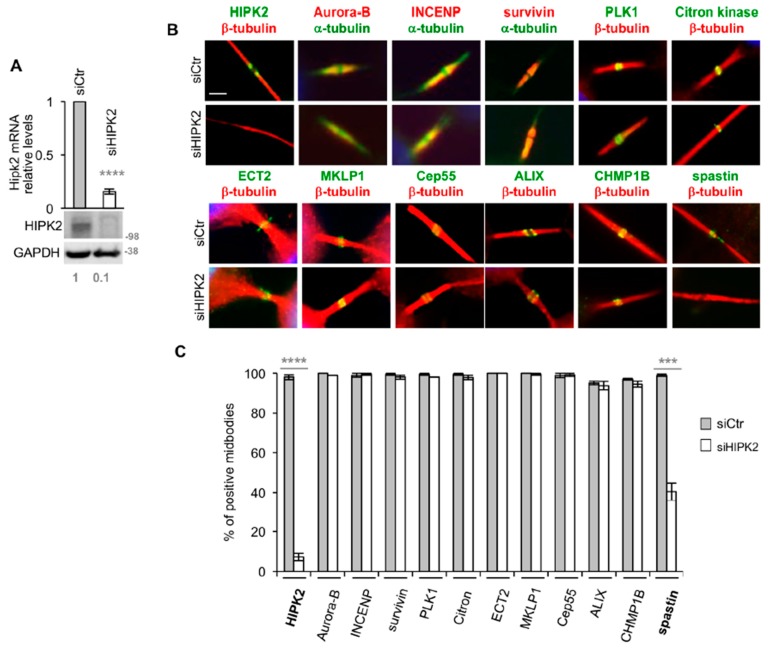
Spastin localization at midbody is reduced in HIPK2-depleted cells. (**A**,**C**) HeLa cells transfected with a mix of three validated HIPK2-specific (siHIPK2) or negative control (siCtr) stealth siRNAs were analyzed by real-time (RT)-PCR, WB and IF 96h post transfection. Molecular weight markers are reported here and in the following figures in kilodalton (kDa). Values are mean ± standard error of mean (SEM) from two independent experiments, each performed in triplicate. ****p < 0.0001, *t*-test. Representative WB is shown and relative densitometric values of HIPK2/GAPDH are reported below each lane. IF was performed with Abs, recognizing indicated proteins in combination with anti-α- or -β-tubulin, to mark midbody and DAPI to stain DNA. In (**B**), representative immunostainings of midbody magnification are shown. Bar, 2 µM. In (**C**), data quantification is shown, values are mean ± SEM from three independent experiments, each performed in duplicate, and >100 midbodies were analyzed per condition. Note that, to avoid alterations of the IF signals, siHIPK2 midbodies showing >2 tubulin-labelled puncta were excluded by this analysis; ***p < 0.001 (*p* = 0.0002), *t*-test.

Thus, we made our analyses upon acute HIPK2-depletion (siHIPK2) obtained by RNAi [22]. IF was performed when HIPK2 mRNA and protein levels were both strongly reduced when compared with control cells (siCtr) (Figure 1A). We observed that HIPK2 depletion does not inhibit the midbody localization of cytokinesis master regulative kinases, such as Aurora B complex and PLK-1, proteins involved in the formation/stabilization of the midbody, such as Citron kinase, MKLP1, and ECT2, and abscission factors, such as Cep55, ALIX, and CHMP1B [25] (Figure 1B,C). During this analysis, the only factor whose localization was strongly impaired by HIPK2 depletion was spastin, which was not detectable at the midbody in a high percentage of siHIPK2 cells (Figure 1B,C). These results suggest that HIPK2 regulates spastin localization at the midbody.

### 3.2. HIPK2-Depleted Cells Show Abscission Defects Similar to Spastin-Depleted Cells

**Figure 2:**
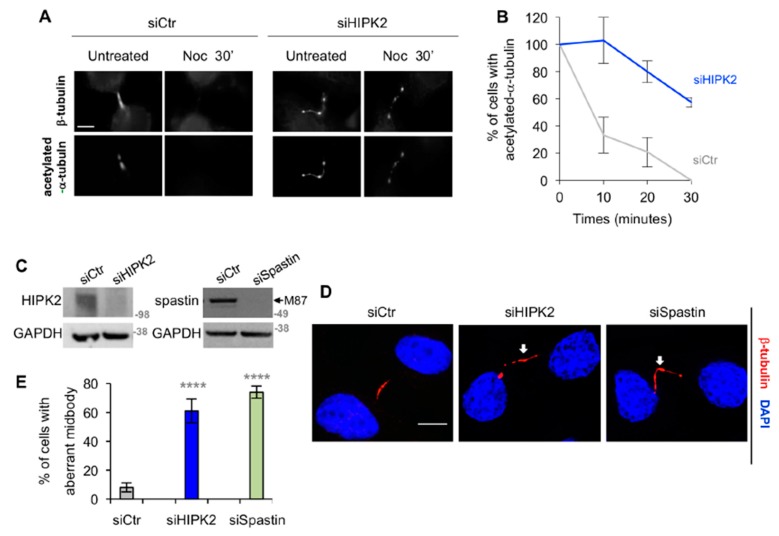
HIPK2-depleted cells show aberrant midbodies. (**A**,**B**) Indicated HeLa cells were analyzed by IF 96 h post transfection at the indicated time post nocodazole (Noc) treatment on ice. Telophase cells, showing acetylated tubulin at the midbody, were scored. In (**A**), representative immunostainings are shown. Bar, 5 µM. In (**B**), the percentage of cells with acetylated tubulin at the midbody are reported as mean ± SEM from two independent experiments, each performed in duplicate, in which at least a total of 80 midbodies per condition were counted. (**C**–**E**) HeLa cells were transfected as in Figure 1A or with spastin-specific validated stealth siRNAs and analyzed 96 h post transfection by WB (**C**) and by IF (**D**,**E**). In (**C**), the arrow indicates the band corresponding to M87, the most abundant spastin form; M87∆4 was generally less abundant and barely detectable. In (**D**), representative immunostainings of siHIP2 and siSpastin telophase cells with aberrant midbody are shown. The arrows indicate tubulin-labeled puncta along the intercellular bridge. Bar, 5 µM. In (**E**), the percentage of cells showing aberrant midbodies is reported as mean ± SEM from three independent experiments, in which at least total 150 midbodies per condition were analyzed. ****p < 0.0001, χ^2^ test.

Next, we evaluated whether siHIPK2 cells show functional defects compatible with the reduction of spastin-positive midbody. Consistent with the spastin role in MT severing and the expectation that loss of spastin results in greater MT stabilization [26], we observed hyperstability of the midbody MT in siHIPK2 cells when analyzed by cold MT depolymerization assay in the presence of nocodazole (Figure 2A,B). Furthermore, we observed that the midbodies of siHIPK2 cells resemble the aberrant phenotype as previously described in spastin-depleted cells, i.e., MT-filled elongated midbodies often associated with tubulin-labelled puncta [5] (Figure 2C–E). From here on, we will generally refer to these phenotypes as “aberrant midbodies”. Comparable results were obtained in HeLa cells depleted of both HIPK2 and spastin (data not shown) in other human HIPK2-depleted cells (Figure S1A) and by transfecting single HIPK2-specific siRNA, ruling out RNAi-off target effects (Figure S1B,C).

These results show a link between abscission defects and the reduction of spastin-positive midbodies in HIPK2-depleted cells, suggesting a role for HIPK2 upstream of spastin.

### 3.3. HIPK2 Phosphorylates Spastin at S268

**Figure 3:**
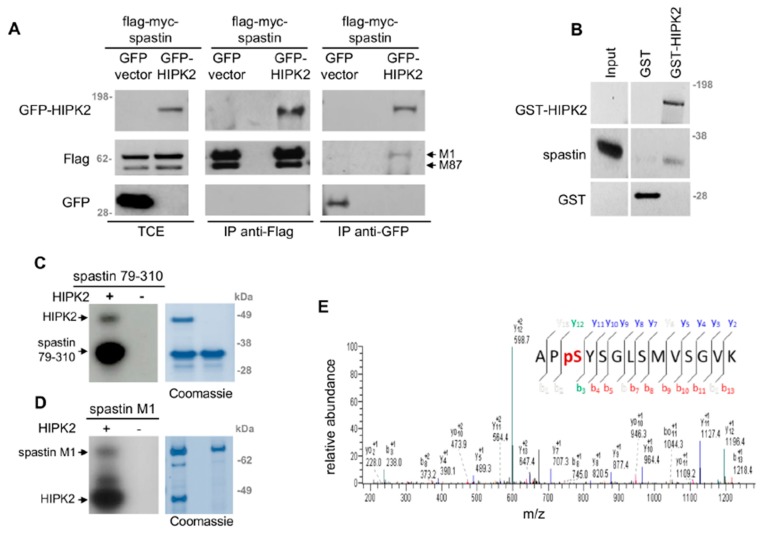
HIPK2 phosphorylates spastin at S268. (**A**) HeLa cells were transfected with the indicated combination of vectors. Exogenous flag-myc-tagged spastin, expressing both M1 and M87 isoforms, was immunoprecipitated with anti-Flag (mouse Ab by OriGene Technologies), or exogenous GFP-tagged HIPK2 was immunoprecipitated with anti-GFP (mouse Ab by Roche). WB for indicated proteins was performed by using anti-Flag (rabbit Ab by Sigma-Aldrich) and anti-GFP (rabbit Ab by Santa Cruz Technology). Different exposure times for TCE and IP are shown. (**B**) GST and GST-HIPK2 proteins were produced by T7-vaccinia system in H1299 cells, purified by GST pull-down, and incubated with an equal amount of recombinant His_6_-tagged spastin 79–310. Spastin binding was detected by WB. Input = 50 ng of recombinant spastin. Representative WB is shown (blot was vertically cropped to eliminate non-related samples). (**C**) In vitro kinase assay was performed by using HIPK2 kinase domain (HIPK2) as the enzymatic source and His_6_-tagged spastin 79–310 as the substrate in presence of γ-ATP-P^32^. Representative autoradiography and relative Coomassie blue staining are shown. (**D**) In vitro kinase assay, performed as in C by using flag-myc-tagged spastin full-length M1 as the substrate. Representative autoradiography and relative Coomassie blue staining are shown. (**D**) In vitro kinase assay, performed as in C, by using cold ATP and analyzed by MS. The ion 731.9, corresponding to the phosphorylated spastin peptide (266–279) encompassing S268, was fragmented, producing a MS2 spectrum with the most intense ion 628.9. This ion revealed a 49 Da neutral loss and was further fragmented in the MS^3^ spectrum that is shown.

Thus, we evaluated whether spastin is a direct target of HIPK2. By co-IP experiments and in vitro assays, we showed that HIPK2 binds and phosphorylates spastin (Figure 3). We co-immunoprecipited epitope-tagged flag-myc-spastin and GFP–HIPK2, by transiently expressing these proteins in HeLa cells and immunoprecipitating with anti-Flag or anti-GFP Abs (Figure 3A). In in vitro assays, HIPK2 was able to bind and phosphorylate a recombinant spastin fusion protein, i.e., a His_6_-tagged fragment spanning aa 79–310 produced in bacteria (Figure 3B,C). In addition, HIPK2 was able to phosphorylate the flag-myc-spastin full-length M1 produced in mammalian cells (Figure 3D). Furthermore, by MS analysis, performed on the recombinant His_6_-tagged spastin in vitro phosphorylated by HIPK2, we identified spastin-S268 as a HIPK2 phosphorylation site (Figure 3E).

S268 is an evolutionary conserved residue (Figure S2A), common to all spastin isoforms, located in a disordered region between the MIT and the MTBD domain. This residue has been reported to be phosphorylated in vivo by large-scale MS analysis performed in human and mouse cells (https://www.phosphosite.org). In addition, it is preceded by a proline, a consensus motif already reported for other HIPK2 targets [27]. Thus, we generated vectors expressing non-phosphorylatable or phosphomimetic spastin derivatives (S268A and S268D, respectively), and produced and validated an Ab that specifically recognizes spastin phosphorylated at S268 (p-S268 Ab; Figure S2B,C).

### 3.4. Spastin-S268 Phosphorylation Is Present at the Midbody and Is Required for Abscission

**Figure 4:**
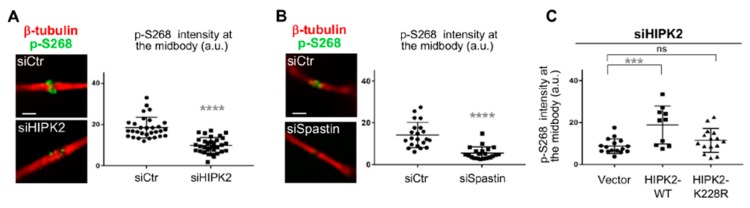
Analysis of spastin-S268 phosphorylation at the midbody. (**A**) Indicated HeLa cells were fixed and stained with DAPI, anti-β-tubulin, and p-S268 Abs, and analyzed to quantify the IF signal of p-S268 spastin at the midbody. Representative immunostainings of midbody magnification are shown. Bar, 1 μM. Quantification in arbitrary units (a.u.) is reported in the dot plot showing the single measures, as well as, the mean value ± SEM from two different experiments ****p < 0.0001, Mann–Whitney test. (**B**) Indicated HeLa cells were stained and analyzed as in A. Representative immunostainings of midbody magnification are shown. Bar, 1 µM. Quantification is reported in the dot plot showing the single measures, as well as, the mean value ± SEM from three different experiments ****p < 0.0001, Mann–Whitney test. (**C**) Dot plot showing the quantification of the p-S268 spastin IF signal in indicated HeLa cells 24 h upon transfection with low doses of vectors expressing GFP-tagged murine HIPK2-WT or its kinase defective derivative. siHIPK2 cells were transfected with indicated vectors 48 h post siRNA transfection performed as in Figure 1A and analyzed 48 h post vector transfection by IF. Note that, in siHIPK2 cells, the expression of murine HIPK2 protein, showing >97% identity with human HIPK2 and fully recapitulating its functions, is not inhibited by RNAi because human-specific siRNAs were used. ***p < 0.001(*p* = 0.0005) *t*-test.

To evaluate whether the midbody localized spastin is phosphorylated at S268, we performed IF with p-S268 Ab. A clear immunostaining was detected at the midbody in the control cells (Figure 4A,B; siCtr panels), while a significant decrease of this staining was observed in siHIPK2 and siSpastin cells, both without spastin at the midbody, confirming the absence of non-specific Ab signals in IF (Figure 4A,B).

To verify the contribution of HIPK2 in spastin-S268 phosphorylation, we restored HIPK2 expression in the siHIPK2 cells by transfection of siRNA-resistant HIPK2 forms. Expression of the HIPK2-WT form reestablished spastin-S268 phosphorylation at the midbody. In contrast, the kinase-defective form, i.e., the HIPK2-K228R mutant, did not restore spastin phosphorylation (Figure 4C), indicating that the HIPK2 kinase activity is required for spastin-S268 phosphorylation at the midbody.

**Figure 5:**
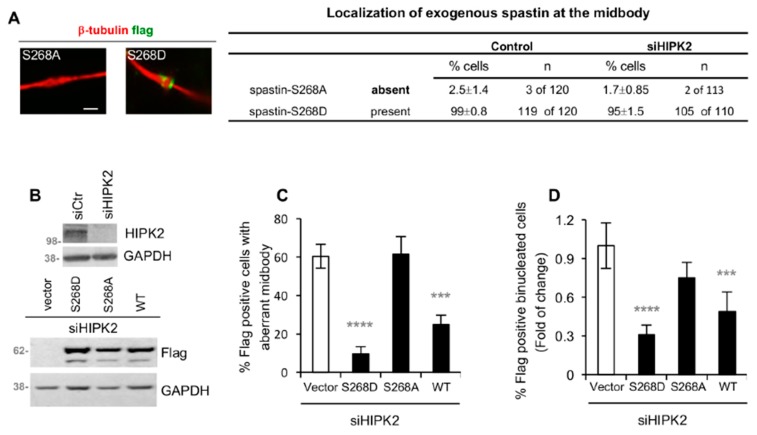
Spastin-S268D restores abscission in siHIPK2 cells. (**A**) HeLa control cells and siHIPK2 cells were transfected with vectors expressing flag-myc-tagged spastin-S268A or -S268D and analyzed 48 h post transfection by IF after staining with DAPI, anti-β-tubulin, and anti-flag Ab to visualize midbody localization of exogenous spastin. Representative immunostainings of midbody magnification are shown in the left panels. Bar, 1 µM. Data quantification are reported, the values are mean ± SEM from three independent experiments. Similar data were obtained by using anti-myc Ab to stain exogenous spastin (data not shown). (**B**–**D**) siHIPK2 cells were transfected with flag-myc empty vector or indicated flag-myc spastin-expressing vectors 48 h post siRNA transfection performed as in Figure 1A and analyzed 48 h post vector transfection by WB and IF after staining with anti-Flag, anti-β-tubulin, and DAPI. In (**B**), representative WB are shown. In (**C**), the percentage of Flag-positive cells, showing aberrant midbodies, is reported as mean ± SEM from three independent experiments, each performed in duplicate, in which at least a total of 80 midbodies per condition were analyzed. In (**D**), the percentage of Flag-positive binucleated cells is reported as fold-change relative to that of vector-transfected cells in two independent experiments, in which total >2000 cells were scored. The percentages of Flag-positive binucleated cells (mean ± SEM) are the following: 4 ± 0.5, 1.95 ± 0.55, 3 ± 0.30, 1.25 ± 0.25 for siHIPK2 cells expressing empty Vector, spastin-WT, -S268D, and -S268A, respectively. ****p < 0.0001, ***p < 0.001(*p* = 0.0002) χ^2^ test.

Next, we evaluated whether this phosphorylation contributes to the cytokinetic localization of spastin by assessing the capability of the non-phosphorylatable spastin-S268A mutant to localize at the midbody. We found that, in contrast with the phosphomimetic S268D forms, spastin-S268A was barely detectable at the midbody (Figure 5A). As expected, the midbody localization of these spastin mutants was independent of HIPK2 (Figure 5A, right columns). Then, we asked whether expression of the phosphomimetic spastin-S268D mutant could rescue the abscission defects of siHIPK2 cells. Microscopy assessment of aberrant midbodies and binucleated cells showed a significant reduction in the percentage of defects, following expression of S268D (Figure 5B–D). A comparable effect was obtained following expression of spastin-WT, even if spastin-S268D resulted more efficient than -WT in these analyses (Figure 5B–D). In contrast, the non-phosphorylatable S268A mutant, according to its impaired midbody localization, was unable to rescue abscission in siHIPK2 cells (Figure 5B–D), although it maintained its enzymatic MT-severing activity (Figure S3A).

These results indicate that HIPK2-mediated S268 phosphorylation of spastin contributes to its midbody localization and activity for abscission.

## 4. Discussion

In this study, we showed that HIPK2 contributes to abscission through phosphorylation of spastin at S268. We observed the presence of phospho-S268 spastin at the midbody and a strong reduction of this spastin localization in HIPK2-depleted cells, associated with cytokinesis defects, which can be rescued by expressing phosphomimetic spastin mutant (i.e., S268D), supporting a key role of HIPK2-mediated spastin phosphorylation for spastin midbody localization to allow for successful abscission.

Thus far, several studies have been carried out to investigate the mechanisms involved in spastin regulation. It has been demonstrated that spastin is regulated by NRF1 and SOX11 transcription factors, by miR-96, miR-182 and Elk1 [28,29]; however, spastin post-translational regulation mechanisms are largely unknown. Notably, we identified a novel regulatory mechanism in which HIPK2 assures spastin midbody presence by phosphorylating it at S268. By MS and phospho-specific Ab we identified HIPK2 as the first kinase that phosphorylates spastin. Even if our analysis did not preclude the existence of other HIPK2 phosphorylation site(s), S268 phosphorylation appears to be crucial for spastin in cytokinesis, as we functionally showed by phosphomimetic and non-phosphorylatable mutants.

At this point, how S268 phosphorylation regulates spastin localization during abscission remains to be clarified. Our single-cell screening shows that HIPK2-depletion impairs the midbody localization of spastin without inhibiting that of its recruiter CHMP1B and of other upstream cytokinesis factors. We can hypothesize that S268 phosphorylation promotes midbody recruitment by stabilizing spastin interaction with its recruiter or other proteins, or induces conformational changes that affect its stability in this subcompartment, by ensuring the concentration of this enzyme at the end of abscission, when its MT-severing function is necessary. By WB analysis performed on TCEs from telophase-enriched cells, we observed a reduction of spastin levels in the HIPK2-depleted cells when compared to their control cells (Figure S3B), indicating that HIPK2 depletion reduces spastin levels, and not only its localization, at the midbody. Furthermore, we obtained similar data on TCE from asynchronous cells (Figure S3C), and we noticed a general decrease of spastin signals by IF in the HIPK2-depleted interphase cells (Figure S3D), suggesting that the HIPK2-mediated regulation of spastin might not be restricted to cytokinesis. Interestingly, katanin, a microtubule-severing enzyme belonging to the same ATPase family of spastin, is regulated via phosphorylation in a disordered protein region similar to that in which S268 is located in spastin [30,31], opening the possibility for a more general mechanism for regulation of AAA ATPases, presenting the same domain arrangement as spastin. Furthermore, both HIPK2 and spastin have been shown to play roles in different biological processes. Our data open the possibility that HIPK2 can also function as a spastin regulator in other spastin-dependent processes, such as neurite branching, axonal growth, endosome tubulation, and nuclear envelope sealing [32].

**Figure 6:**
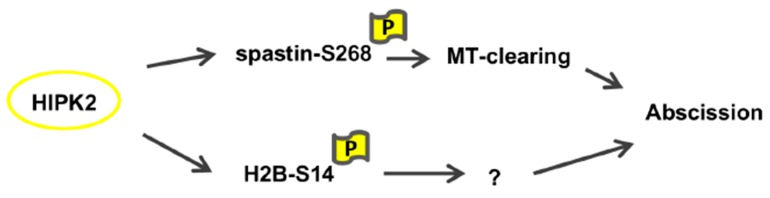
Schematic representation of HIPK2 activity on spastin and H2B during cytokinesis.

Previously, we have demonstrated that the expression of the phosphomimetic mutant H2B-S14D can restore successful cytokinesis in HIPK2-depleted cells [22,23]. We observed that the abscission defects were rescued in siHIPK2 cells expressing H2B-S14D without affecting S268 spastin phosphorylation at the midbody (pS268 intensity at the midbody was 12.9 ± 8.0 and 11.7 ± 5.3 in siHIPK2 and in siHIPK2+H2B-S14D cells, respectively). Similarly, these defects were rescued in siHIPK2 cells expressing spastin-S268D, independently of H2B-S14 phosphorylation (pH2B-S14 intensity at the midbody was 5.1 ± 2.9 and 4.9 ± 2.7 in siHIPK2 and in siHIPK2 cells+spastin-S268D, respectively). These observations suggest two parallel mechanisms of action of HIPK2 in cytokinesis (Figure 6). Further supporting this idea, depletion of extrachromosomal H2B had no effect on the presence of spastin at the midbody (LM and SS, manuscript in preparation). Thus, similar to other cytokinesis regulating kinases [33,34], HIPK2 contributes to cytokinesis by acting on multiple targets on different pathways.

## Supplementary Materials

The following are available online at https://www.mdpi.com/2073-4409/8/7/684/s1, **Figure S1**. Aberrant midbodies after HIPK2 depletion; **Figure S2.** pS268 Ab validation; **Figure S3.** Spastin-S268A MT severing activity and spastin levels in HIPK2-depleted cells. **TableS1:** List of Abs and IF conditions.

## References

[1] Mierzwa B., Gerlich D.W. (2014). Cytokinetic abscission: Molecular mechanisms and temporal control. Dev. Cell.

[2] Glotzer M. (2017). Cytokinesis in Metazoa and Fungi. Cold Spring Harb. Perspect. Biol..

[3] Stoten C.L., Carlton J.G. (2018). ESCRT-dependent control of membrane remodelling during cell division. Semin. Cell Dev. Biol..

[4] Yang D., Rismanchi N., Renvoisé B., Lippincott-Schwartz J., Blackstone C., Hurley J.H. (2008). Structural basis for midbody targeting of spastin by the ESCRT-III protein CHMP1B. Nat. Struct. Mol. Biol..

[5] Connell J.W., Lindon C., Luzio J.P., Reid E. (2009). Spastin couples microtubule severing to membrane traffic in completion of cytokinesis and secretion. Traffic.

[6] Claudiani P., Riano E., Errico A., Andolfi G., Rugarli E.I. (2005). Spastin subcellular localization is regulated through usage of different translation start sites and active export from the nucleus. Exp. Cell Res..

[7] D’Avino P.P., Capalbo L. (2016). Regulation of midbody formation and function by mitotic kinases. Semin. Cell Dev. Biol..

[8] Nähse V., Christ L., Stenmark H., Campsteijn C. (2017). The Abscission Checkpoint: Making It to the Final Cut. Trends Cell Biol..

[9] Mancuso G., Rugarli E.I. (2008). A cryptic promoter in the first exon of the SPG4 gene directs the synthesis of the 60-kDa spastin isoform. BMC Biol..

[10] Solowska J., Garbern J., Baas P.W. (2010). Evaluation of loss-of-function as an explanation for SPG4-based hereditary spastic paraplegia. Hum. Mol. Genet..

[11] Guizetti J., Schermelleh L., Mäntler J., Maar S., Poser I., Leonhardt H., Müller-Reichert T., Gerlich D.W. (2011). Cortical constriction during abscission involves helices of ESCRT-III-dependent filaments. Science.

[12] Goliand I., Adar-Levor S., Segal I., Nachmias D., Dadosh T., Kozlov M.M., Elia N. (2018). Resolving ESCRT-III Spirals at the Intercellular Bridge of Dividing Cells Using 3D STORM. Cell Rep..

[13] Allison R., Lumb J.H., Fassier C., Connell J.W., Ten Martin D., Seaman M.N., Hazan J., Reid E. (2013). An ESCRT-spastin interaction promotes fission of recycling tubules from the endosome. J. Cell Biol..

[14] Papadopoulos C., Orso G., Mancuso G., Herholz M., Gumeni S., Tadepalle N., Jüngst C., Tzschichholz A., Schauss A., Höning S. (2015). Spastin binds to lipid droplets and affects lipid metabolism. PLoS Genet..

[15] Errico A., Claudiani P., D’Addio M., Rugarli E.I. (2004). Spastin interacts with the centrosomal protein NA14, and is enriched in the spindle pole, the midbody and the distal axon. Hum. Mol. Genet..

[16] Ventimiglia L.N., Cuesta-Geijo M.A., Martinelli N., Caballe A., Macheboeuf P., Miguet N., Parnham I.M., Olmos Y., Carlton J.G., Weissenhorn W. (2018). CC2D1B Coordinates ESCRT-III Activity during the Mitotic Reformation of the Nuclear Envelope. Dev. Cell.

[17] Saul V.V., de la Vega L., Milanovic M., Krüger M., Braun T., Fritz-Wolf K., Becker K., Schmitz M.L. (2013). HIPK2 kinase activity depends on cis-autophosphorylation of its activation loop. J. Mol. Cell Biol..

[18] Siepi F., Gatti V., Camerini S., Crescenzi M., Soddu S. (2013). HIPK2 catalytic activity and subcellular localization are regulated by activation-loop Y354 autophosphorylation. Biochim. Biophys. Acta.

[19] Scaglione A., Monteonofrio L., Parisi G., Cecchetti C., Siepi F., Rinaldo C., Giorgi A., Verzili D., Zamparelli C., Savino C. (2018). Effects of Y361-auto-phosphorylation on structural plasticity of the HIPK2 kinase domain. Protein Sci..

[20] D’Orazi G., Rinaldo C., Soddu S. (2012). Updates on HIPK2: A resourceful oncosuppressor for clearing cancer. J. Exp. Clin. Cancer Res..

[21] Blaquiere J.A., Verheyen E.M. (2017). Homeodomain-Interacting Protein Kinases: Diverse and Complex Roles in Development and Disease. Curr. Top. Dev. Biol..

[22] Rinaldo C., Moncada A., Gradi A., Ciuffini L., D’Eliseo D., Siepi F., Prodosmo A., Giorgi A., Pierantoni G.M., Trapasso F. (2012). HIPK2 controls cytokinesis and prevents tetraploidization by phosphorylating histone H2B at the midbody. Mol. Cell.

[23] Valente D., Bossi G., Moncada A., Tornincasa M., Indelicato S., Piscuoglio S., Karamitopoulou E.D., Bartolazzi A., Pierantoni G.M., Fusco A. (2015). HIPK2 deficiency causes chromosomal instability by cytokinesis failure and increases tumorigenicity. Oncotarget.

[24] Monteonofrio L., Valente D., Ferrara M., Camerini S., Miscione R., Crescenzi M., Rinaldo C., Soddu S. (2018). HIPK2 and extrachromosomal histone H2B are separately recruited by Aurora-B for cytokinesis. Oncogene.

[25] Hu C.K., Coughlin M., Mitchison T.J. (2012). Midbody assembly and its regulation during cytokinesis. Mol. Biol. Cell.

[26] Riano E., Martignoni M., Mancuso G., Cartelli D., Crippa F., Toldo I., Siciliano G., Di Bella D., Taroni F., Bassi M.T. (2009). Pleiotropic effects of spastin on neurite growth depending on expression levels. J. Neurochem..

[27] Cecchinelli B., Porrello A., Lazzari C., Gradi A., Bossi G., D’Angelo M., Sacchi A., Soddu S. (2006). Ser58 of mouse p53 is the homologue of human Ser46 and is phosphorylated by HIPK2 in apoptosis. Cell Death Differ..

[28] Henson B.J., Zhu W., Hardaway K., Wetzel J.L., Stefan M., Albers K.M., Nicholls R.D. (2012). Transcriptional and post-transcriptional regulation of SPAST, the gene most frequently mutated in hereditary spastic paraplegia. PLoS ONE.

[29] Canbaz D.M., Kırımtay K., Karaca E., Karabay A. (2011). SPG4 gene promoter regulation via Elk1 transcription factor. J. Neurochem..

[30] Maddika S., Chen J. (2009). Protein kinase DYRK2 is a scaffold that facilitates assembly of an E3 ligase. Nat. Cell Biol..

[31] Whitehead E., Heald R., Wilbur J.D. (2013). N-terminal phosphorylation of p60 katanin directly regulates microtubule severing. J. Mol. Biol..

[32] McNally F.J., Roll-Mecak A. (2018). Microtubule-severing enzymes: From cellular functions to molecular mechanism. J. Cell Biol..

[33] Afonso O., Figueiredo A.C., Maiato H. (2017). Late mitotic functions of Aurora kinases. Chromosoma.

[34] Combes G., Alharbi I., Braga L.G., Elowe S. (2017). Playing polo during mitosis: PLK1 takes the lead. Oncogene.

