# Supplementary material for: HIPK2 Phosphorylates the Microtubule-Severing Enzyme Spastin at S268 for Abscission
Source: Cells. 2019 Jul 5;8(7):684. doi: 10.3390/cells8070684 (PMC6678495; doi:10.3390/cells8070684)
Supplement: Supplementary file 1 [file cells-08-00684-s001.pdf]

Supplementary Table 1: List of Abs and IF conditions. F/P= Fixation and permeabilization were performed in combination as reported in [22]. RT = Room Temperature; O/N = overnight.

**Primary Ab** (supplier)	**Fixation**			**permeabilization**	**Dilution, incubation time and temperature**	**Specie**
	Methanol 5′ −20°C	Formaldehyde 10′ RT	F/P	Triton 0,25% 10′ RT		
**ALIX** (Santa Cruz Biotechnoloy.)	X			X	1:100 2h RT	Mouse
**Cep55** (Abnova)		X		X	1:700 2h RT	Mouse
**AURORA-B** (BD bioscience)		X			1:100 1h RT	Mouse
**INCENP** (Abcam)		X			1:500 1h RT	Mouse
**Survivin** (Epitomics)		X		X	1:100 1h RT	Rabbit
**ECT-2** (Santa Cruz Biotechnology)	X			X	1:100 2h RT	Rabbit
**MKLP-1** (Santa Cruz Biotechnology)	X			X	1:100 2h RT	Rabbit
**HIPK2 946** (self-made [22])	X			X	1:100 O/N. +4°C	Rabbit
**Sp3G11/1 SPASTIN** (Santa Cruz Biotechnology)	X			X	1:100 2 h RT	Mouse
**PLK1** (Santa Cruz Biotechnology)		X		X	1: 100 2h RT	Mouse
**Citron kinase** (Dr. Di Cunto's kind gift)	X			X	1:100 2 h RT	Mouse
**CHMP1B** **(**Abnova)		X	X	X	1:500 2h RT	mouse
**p-S268 spastin** (self-made, this paper)	X			X	1:100 2h RT	Rabbit

**Flag** (Sigma-Aldrich)	X		X	1:100 2h RT	Rabbit
**α tubulin FITC** (Sigma-Aldrich)	X	X	X	1:200 1h 37°C	Mouse
**β-Tubulin-Cy3** (Sigma-Aldrich)	X	X	X	1:200 1h 37°C	Mouse
**Acetyl-α-tubulin** (Sigma-Aldrich)	X		X	1:200 2h RT	Rabbit

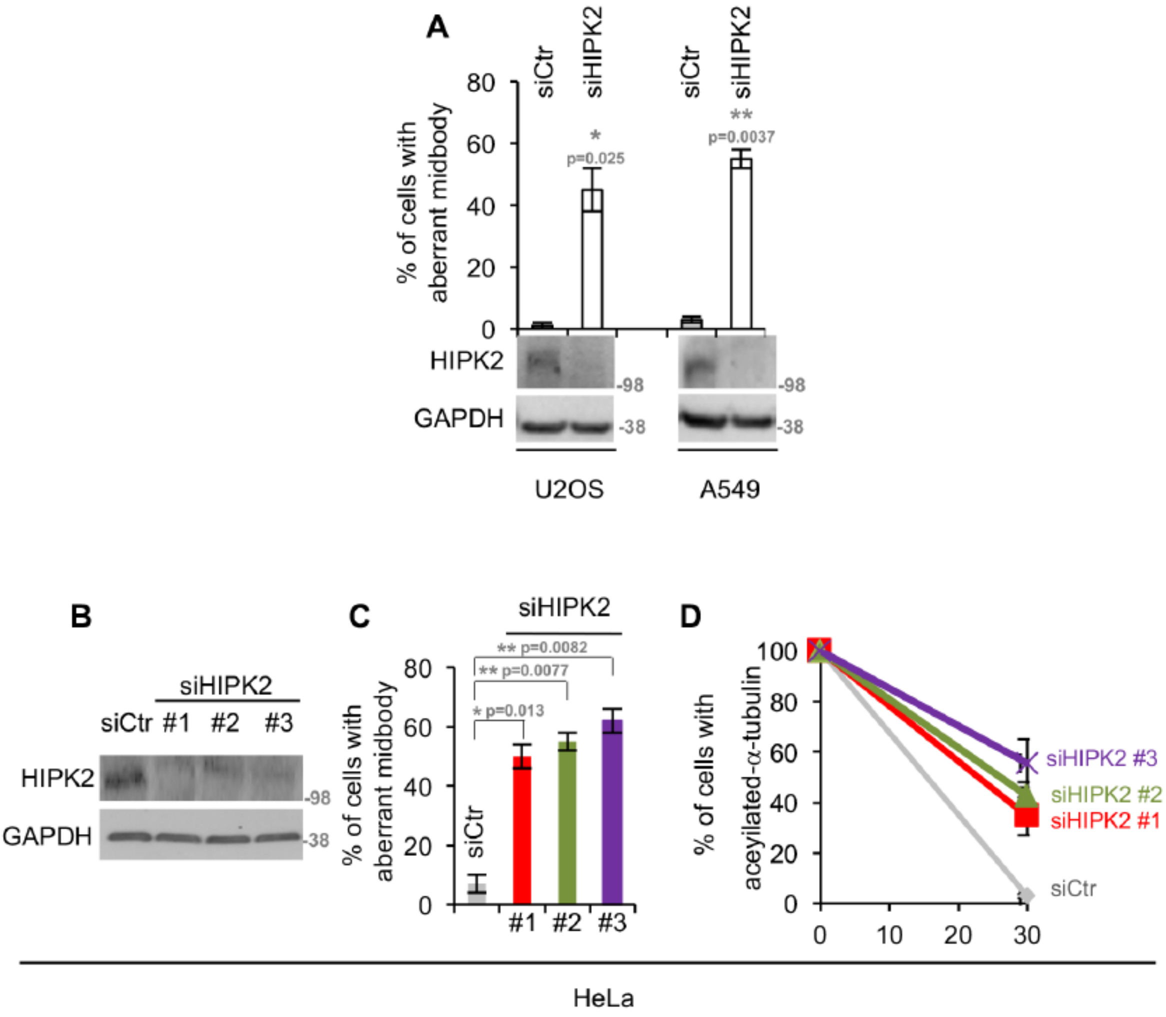

Figure S1. Aberrant midbodies after HIPK2 depletion**. (A)** Indicated cells were transfected as in 1A and analyzed 96h post transfection by WB and IF as in 2E. The percentage of cells showing aberrant midbodies is reported as mean ± SEM from 2 independent experiments; in which at least total 100 midbodies per condition were analyzed. *p < 0.05, **p <0.01, t-test. Representative WB are shown below the chart. (**B**–**C**) HeLa cells were transfected with the single indicated HIPK2-specific siRNAs and analyzed 96h post transfection by WB and IF; siCtr cells were used as control. In B, representative WB is shown. In C, IF was performed as in 2E and the percentage of cells showing aberrant midbodies is reported as mean ± SEM from 2 independent experiments; in which at least total 100 midbodies per condition were analyzed. p < 0.05, ** p < 0.01, t-test. In **D**, IF was performed after nocodazole treatment on ice performed as in 2A for 30 min, the percentage of cells with acetylated tubulin at the midbody are reported as mean ± SEM from 2 independent experiments, each performed in duplicate, in which at least total 80 midbodies per condition were counted.

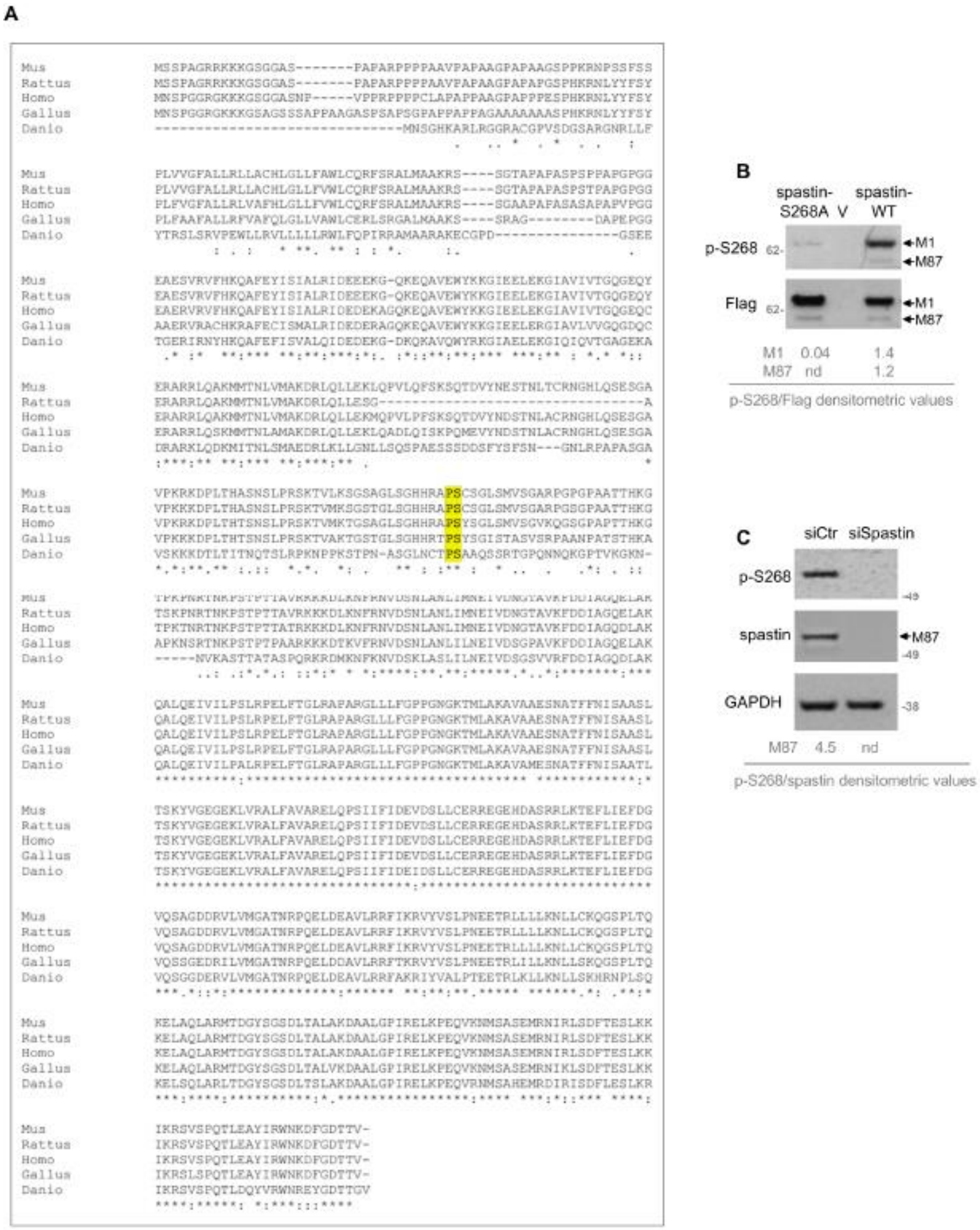

Figure S2. pS268 Ab validation. (**A**) ClustalW alignment of the full length spastin sequences, the human S268 preceded by a proline residue (P) and analogous residues are highlighted. (**B**) HeLa cells were transfected with vectors expressing flag-myc tagged spastin-S268A, flag-myc empty vector (V), or flag-myc tagged spastin-WT and analysed by WB 24h post transfection using anti-pS268 and anti-Flag (mouse by OriGene Technologies) Abs. Representative WB is shown. (**C**) HeLa cells were transfected as in 2C with spastin-specific siRNA and analysed 96h post transfection by WB with indicated Abs. Representative WB is shown. The ratio of the densitometric values of the indicated bands are reported below each lane; nd = not determinable.

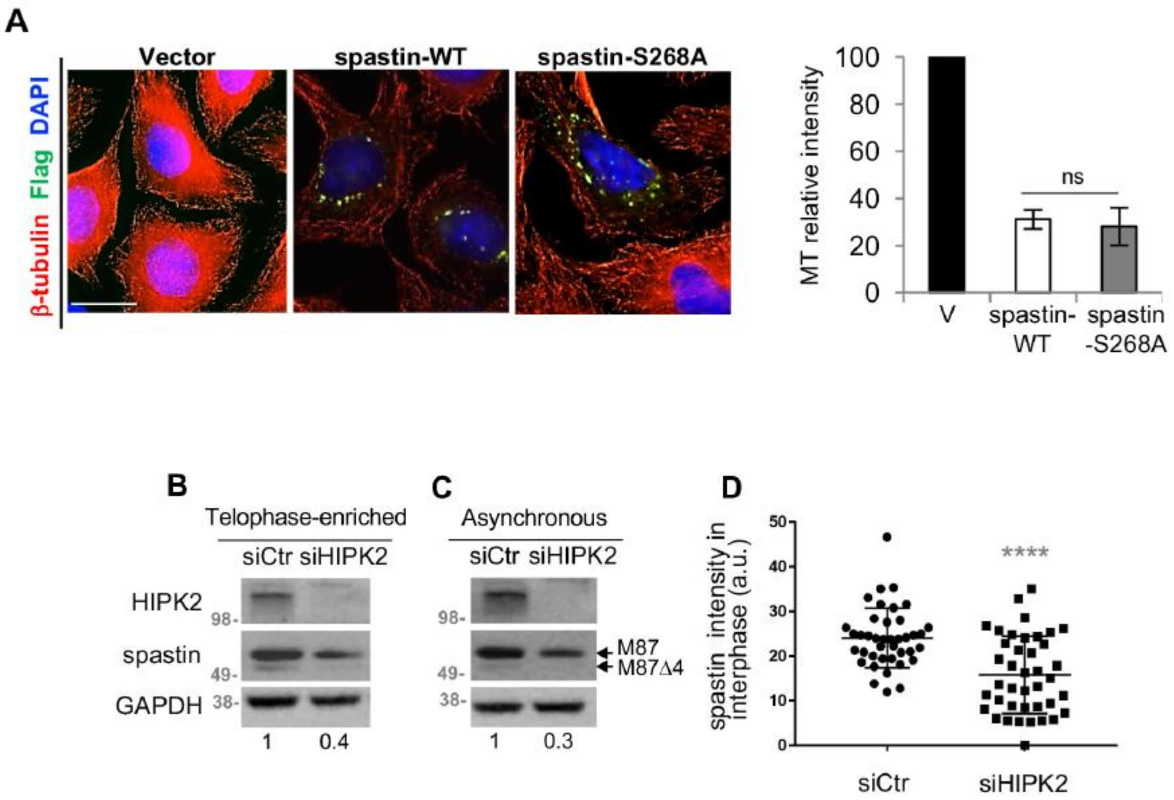

Figure S3. Spastin-S268A MT severing activity and spastin levels in HIPK2-depleted cells. **(A)** HeLa cells were transfected with flag-myc tagged spastin-S268A or spastin-WT expressing vectors. Cells were analyzed by IF 24h post transfection after staining with anti-Flag, anti-β-tubulin and DAPI. Cells expressing flag-myc vector were used as control. Representative immunostainings are shown. Quantification of MT density by measuring β-tubulin fluorescence intensity for at least 30 cells per sample is reported; values are mean ± SEM, relative to the mean intensity of control cells. For this analysis, we selected cells expressing similar levels of spastin-WT and -S268A. Bar, 10 µM. Similar levels of MT loss are present in cells expressing spastin-WT and -S268A, indicating that S268A mutation does not affect the severing activity of spastin. (**B**–**D**) HeLa cells were transfected as in 1A. Telophase-enriched and asynchronous cells were analyzed by WB with the indicated Abs 96h post transfection. The efficiency of telophase enrichment (≈70%) was similar in siCtr and siHIPK2 cells. Representative WB are shown in B and in C, relative densitometric values of spastin M87/GAPDH are reported below each lane. In D, asynchronous siCtr and siHIPK2 cells were analyzed 4d post transfection by IF after staining with anti-spastin, anti-β-tubulin and DAPI. Quantification of spastin IF signals in interphase cells is reported in arbitrary units (a.u.) in the dot plot showing the single measures, as well as, the mean value ± SEM from 2 different experiments. The fluorescence intensity of spastin was obtained by drawing cellular outline and measuring the mean of pixel intensity corrected for external background ****$p < 0.0001$, t-test.
